# Editorial: Women in applied genetic epidemiology

**DOI:** 10.3389/fgene.2024.1508169

**Published:** 2024-11-26

**Authors:** Qi Guo, Kristina Allen-Brady, Marta Olszewska

**Affiliations:** ^1^ Insmed Innovation UK, Cambridge, United Kingdom; ^2^ Department of Internal Medicine, University of Utah, Salt Lake City, UT, United States; ^3^ Institute of Human Genetics, Polish Academy of Sciences, Poznan, Poland

**Keywords:** protein-truncating variants, cellular component, breast cancer susceptibility, panel sequencing, RNA-seq, cardiovascular disease, women’s reproductive health

Genetic epidemiology is a multidisciplinary field that aims to understand the role of genetic factors in the distribution and determinants of health and disease in populations. By applying statistical and epidemiological methods to genetic data, this field seeks to identify genetic variations that contribute to disease risk, susceptibility, and the inheritance of traits. The insights gained through genetic epidemiology are crucial for uncovering the genetic basis of complex diseases and for advancing personalized medicine, prevention strategies, and targeted therapies.

The Research Topic “Women in Genetic Epidemiology” highlights the contributions of women scientists who are advancing the field of genetic epidemiology through diverse research efforts. This Research Topic of papers not only showcases new developments and innovative work led by women across various disciplines within genetic epidemiology but also underscores the importance of gender diversity in driving scientific discovery. This Research Topic comprises one brief research report and five original research articles ranging from association analysis to identification of protein-truncating and rare missense variants in diverse populations, and causal analysis of risk factors on various diseases such as cardiovascular disease and women’s reproductive health.

Keratoconus (KTCN) is a complex, multifactorial condition influenced by genetic, environmental, and lifestyle factors. Building on their prior research, Nowak-Malczewska et al. identified distinct expression patterns in KTCN-related pre-miRNAs through RNA-seq analysis of corneal samples from patients and controls. Their findings showed six miRNAs with notably increased expression and four that were downregulated in KTCN samples. Among these, miR-184 exhibited the largest differential expression between affected and unaffected corneas. These dysregulated miRNAs likely contribute to KTCN pathogenesis by disrupting extracellular matrix organization and signal transduction pathways, aligning with mechanisms previously implicated in the disease.

Recent gene panel testing in large population-based case-control studies has enhanced the accuracy of estimating the prevalence and breast cancer risk associated with pathogenic variants. Zanti et al. leveraging panel sequencing data from the Breast Cancer Association Consortium’s BRIDGES project, examined over 2,000 samples from the MASTOS study—a population-based case-control study on breast cancer in Cyprus. Their analysis found that protein-truncating variants in *BRCA2* and *ATM* were linked to a significantly increased risk of breast cancer, while PTVs in *BRCA1* and *PALB2* specifically elevated the risk for estrogen receptor—negative breast cancer. Additionally, for individuals with a family history of breast cancer, PTVs in *ATM*, *BRCA2*,* BRCA1*, *PALB2*, and *RAD50* correlated with a higher risk. The study reinforced the importance of family history, age at diagnosis, and tumor subtype for effective risk stratification in the general Cypriot population.

Mendelian randomization (MR) is a powerful epidemiological method that uses genetic variants as instrumental variables to assess the causal relationships between risk factors and health outcomes. It leverages the principle that genetic variants are randomly assorted at conception, mimicking the effects of a randomized controlled trial. MR studies are particularly valuable in genetic epidemiology for identifying causal relationships in situations where traditional observational studies may be prone to bias. This approach has been applied to a variety of health conditions, including the 4 MR studies featured within this Research Topic, each contributing insights into their respective areas.

Rukh et al. investigated the association between a derived measure for wellbeing with cardiovascular disease risk (i.e., atrial fibrillation, heart failure, myocardial infarction, and ischemic stroke) using two-sample MR. The genetically predicted wellbeing measure was causally associated with reduction in both heart failure and myocardial infarction. The wellbeing association was found to be mainly driven by depressive symptoms. Their results suggest that improving psychological wellbeing may help in reducing the burden of cardiovascular disease.

As several epidemiologic studies have suggested that smoking initiation, alcohol and coffee consumption are associated with adverse female reproductive health, Jiang et al. in a two-sample MR investigated reproductive hormones and menstruation phenotypes. They found that smoking had a significant causal association with lower sex hormone-binding globulin levels and earlier age at menopause. They further observed suggestive evidence that alcohol consumption was associated with lower total testosterone levels and earlier age at menopause. Coffee consumption did not show an association with any of the female reproductive variables examined. As males were included in the analyses, further research is needed to understand the role of smoking initiation, alcohol and coffee consumption in female populations.

Liu et al. used two-stage MR to investigate a previously observed association between endometriosis and gut microbiota. *Anaerotruncus*, *Desulfovibrio*, *Haemophilus*, and *Holdemania* showed causal association with endometriosis. *Holdemania* and Ruminococcaceae UCG002 exert reversible, stage-specific impacts. For mild endometriosis, the study team identified increased causal associations with *Eubacterium brach* group, *Family_XIII AD3011* group, and Ruminococcaceae *UCG002*. For moderate to severe endometriosis, *Bacteroides*, *Holdemania*, and Lachnospiraceae *NK4A136* groups were identified as protective factors. As specific gut microbiota exhibit causal effects on endometriosis and specific endometriosis stages, opportunities exist for developing new treatments for endometriosis.

As the causes of primary dysmenorrhea are not fully understood and with a goal to gain novel insights into the clinical management of dysmenorrhea, Li et al. investigated the causal link between phosphatidylcholine (PC), a phospholipid involved in biological membranes, and dysmenorrhea using two-sample MR. They found a significant positive association between increased PC levels and dysmenorrhea and suggested that together with its metabolite lysoPC, these molecules may play a regulative role in uterine smooth muscle contractions through a signaling pathway linked to lipid metabolism.

Each of these papers represents a unique contribution to the field, and together they illustrate the impact of women in genetic epidemiology. In addition to showcasing science, this Research Topic celebrates the achievements of women in a field that, like many others, has historically faced gender disparities. By providing a platform for women scientists to share their work, we aim to foster a more inclusive scientific community that values the contributions of all researchers, regardless of gender.

